# Dementia Risk among Coronavirus Disease Survivors: A Nationwide Cohort Study in South Korea

**DOI:** 10.3390/jpm11101015

**Published:** 2021-10-09

**Authors:** Hye-Yoon Park, In-Ae Song, Tak-Kyu Oh

**Affiliations:** 1 Department of Psychiatry, Seoul National University Hospital, Seoul 03080, Korea; psychepark@gmail.com; 2 Department of Psychiatry, Seoul National University College of Medicine, Seoul 03080, Korea; 3 Department of Anesthesiology and Pain Medicine, Seoul National University Bundang Hospital, Seongnam 13620, Korea; songoficu@outlook.kr; 4 Department of Anesthesiology and Pain Medicine, Seoul National University College of Medicine, Seoul 03080, Korea

**Keywords:** COVID-19, COVID-19 survivors, dementia, Alzheimer’s disease, vascular dementia

## Abstract

We aimed to investigate whether coronavirus disease (COVID-19) survivors were at a higher risk of dementia diagnosis compared to controls at 6 months follow-up. Data pertaining to the period between 1 January and 4 June 2020, were extracted from the National Health Insurance Service (NHIS)-COVID-19 database in South Korea. Data on adults (≥20 years old) with no history of dementia, obtained from the NHIS-COVID-19 database, were included in the study. The endpoint of this study was the development of dementia, which was evaluated from 1 January to 1 December 2020. A total of 306,577 adults were included in the analysis, comprising 7133 COVID-19 survivors and 299,444 individuals in the control group. Among the subjects, new-onset dementia diagnosed in 2020 was recorded in 1.2% (3546 of 306,577). In the covariate-adjusted multivariable Cox regression model, the incidence of dementia among COVID-19 survivors was 1.39-fold higher (hazard ratio: 1.39, 95% confidence interval: 1.05–1.85; *p* = 0.023) than that in the control group. At approximately 6 months of follow-up, COVID-19 survivors were at a higher risk of dementia compared to other populations in South Korea.

## 1. Introduction

The coronavirus disease (COVID-19) was declared a pandemic by the World Health Organization on 11 March 2020 [1]. Approximately one year later, as of 30 March 2021, there were 127,581,652 COVID-19 cases and 2791,072 COVID-19-related deaths globally [2]. Although vaccination began on 8 December 2020 [3,4], limitations with respect to the volume of vaccine production and the rapidity of administration presently hamper the development of herd immunity to COVID-19. Therefore, COVID-19 will continue to be the most important and serious global health issue in 2021.

Considering that 2.2% of all individuals diagnosed with COVID-19 have died due to the infection worldwide [2], there might be about 97.8% of COVID-19 survivors currently. As COVID-19 survivors have been reported to suffer from various sequelae after hospitalization [5,6], their quality of life has emerged as a leading public health issue. One of the most important sequalae in this population is dementia, which is one of the leading causes of death and disability and a global health crisis [7]. 

The pathogenic virus, severe acute respiratory syndrome coronavirus-2 (SARS-CoV-2), is known to invade the central nervous system (CNS) [8,9] and can cause various neuropsychiatric complications, including dementia, in patients with COVID-19 during hospitalization [10,11]. Recently, underlying dementia was found to increase the risk of COVID-19 infection in the United States population and this association was more evident for vascular dementia (VD) than Alzheimer’s disease (AD) [12]. Moreover, dementia was reported to be a predictive factor for mortality among COVID-19 patients [13]. However, previous studies have only focused on the impact of dementia on the risk of infection and mortality in either the general adult population or in patients with COVID-19 [10,11,12,13]; no study has evaluated the risk of new-onset dementia in COVID-19 survivors so far. Previous studies reported that COVID-19 caused short-term and long-term cognitive deficits in COVID-19 survivors [14,15], which might have also lead to a dementia diagnosis. Moreover, stroke was also common in COVID-19 patients [16] and it also increased dementia prevalence among COVID-19 survivors because stroke is a significant risk factor for dementia [17].

Therefore, we aimed to investigate whether COVID-19 survivors were at a higher risk of dementia diagnosis compared to those without a history of COVID-19 infection. We hypothesized that COVID-19 survivors were associated with a higher risk of dementia diagnosis. 

## 2. Materials and Methods

### 2.1. Study Design and Ethical Concerns

As the current study was a population-based cohort study, we followed the guidelines of the Strengthening the Reporting of Observational Studies in Epidemiology, recommended for epidemiological cohort studies [18]. The deliberation of the study protocol was exempted by the Institutional Review Board (IRB) of Seoul National University Bundang Hospital (X-2009-636-902) and the National Health Insurance Service (NHIS) data sharing service approved the use of data from the NHIS-COVID-19 cohort database (DB) (NHIS-2021-1-070). The requirement for informed consent was waived by the IRB since data were extracted retrospectively in an anonymized form.

### 2.2. Data Source (NHIS-COVID-19 DB)

The NHIS-COVID-19 DB was created for the purpose of medical research in cooperation with the Korea Disease Control and Prevention Agency (KDCPA) and NHIS. The KDCPA provided the data of all individuals infected with COVID-19 confirmed by polymerase chain reaction (PCR) test, between 1 January 2020 and 4 June 2020. The data included patient demographics, treatment results, duration of isolation and the confirmation date of COVID-19 infection by PCR. Meanwhile, the control population was extracted by stratified random sampling of the NHIS DB, which contains data from the South Korean general population. For the stratified random sampling, the age, sex and place of residence of COVID-19 patients were used as strata. The NHIS DB initially divides the general population, other than COVID-19 patients and PCR-negative individuals, into mutually exclusive strata (age, sex and place of residence). Next, 15 more samples are drawn from each of the strata with sizes that are proportional to the strata’s in the target population (COVID-19 patients). Through this stratified random sampling, the control population was considered as a comparative cohort that was similar to the exposed group (COVID-19 patients) with regards to age, sex and place of residence. The stratified random sampling was performed using SAS version 9.4 (SAS Institute, Cary, NC, USA) [19]. Finally, the NHIS-COVID-19 DB also contained the data of individuals who had undergone a COVID-19 PCR test but had tested negative. Thus, the NHIS COVID-19 DB included three groups: the COVID-19 positive group, a control population and individuals who had tested negative. In South Korea, the KDCPA conducted COVID-19 tests for individuals who had direct or indirect contact with COVID-19 patients in the community or hospital, after extensive contact tracing of COVID-19 patients [20]. The detailed information on the medical history of patients from 2015 to 2020, including diagnoses of any underlying diseases coded as per the International Classification of Diseases (ICD)-10 and drug history, were also collected from the NHIS COVID-19 DB. 

### 2.3. Management of COVID-19 Patients in South Korea

In South Korea, COVID-19 patients with severe symptoms such as pneumonia are admitted to the hospital. However, those who have mild or no symptoms are isolated and closely monitored in specific government-managed centers. All COVID-19 patients are isolated by the government until hospital treatment for COVID-19 or until they recover from COVID-19, as confirmed by a negative PCR test. The KDCPA followed up all individuals who were confirmed as COVID-19-positive by PCR until death or recovery from COVID-19 (negative PCR test). After discharge from the hospital or recovery from COVID-19, data regarding the patients’ healthcare resource utilization and diagnoses (including diagnosis of dementia) were registered in the NHIS DB, which is the sole public health insurance service in South Korea.

### 2.4. COVID-19 Survivors and the Control Group (Adults Who Did Not Contract COVID-19)

COVID-19 survivors were defined as individuals diagnosed with COVID-19 and discharged from the hospital after treatment. In addition, COVID-19 patients who were not admitted to the hospital due to mild or no symptoms were defined as COVID-19 survivors once they recovered from COVID-19, as confirmed by a negative PCR test. Among COVID-19 patients, those who received ongoing in-hospital treatment at the time of this study were excluded from the NHIS-COVID-19 DB because treatment results had not yet been determined. To focus on new-onset dementia diagnosed in 2020, we excluded individuals diagnosed with dementia from 1 January 2015, to 31 December 2019. In addition, the control group in this study comprised the control population (stratified random sample) and PCR test-negative individuals, because a larger population was required for a more robust study and to validate our main findings. Therefore, the control group included adults who did not contract COVID-19 in this study. All individuals aged <20 years were excluded from the analysis because the pathophysiology of dementia in children differs from that in the elderly [21].

### 2.5. Endpoint: Development of Dementia

The primary endpoint of this study was the development of dementia, evaluated from 1 January 2020, until 1 December 2020. As for the corresponding ICD-10 codes, dementia included AD (F00, G30), VD (F01) and unspecified dementia (F03); however, dementia due to another causative disease (F02) was excluded. To capture cases of dementia missed by the use of ICD-10 codes in the NHIS, we also extracted the prescription data for acetylcholinesterase inhibitors (AChEIs), since AChEIs are the only drugs developed for the treatment of early dementia [22]. If patients with dementia have a score of ≤26 on the Mini Mental State Exam, the NHIS pays for the cost of AchEIs. Therefore, individuals who had been prescribed AchEIs in the absence of registered ICD-10 codes pertaining to dementia were also considered to have dementia. In South Korea, the data of all patients with dementia, regardless of its type, are registered in the NHIS DB by the physician or psychiatrist so that they may receive financial support for treatment expenses. 

### 2.6. Measurement of Confounders

The following clinicopathological variables were collected as confounders. Age and sex were collected as demographic variables, while the residence and annual income levels in February 2020 were collected as socioeconomic status-related information. Participants were divided into seven groups according to age (20–29, 30–39, 40–49, 50–59, 60–69, 70–79 and ≥80 years) and five groups according to residence (Seoul, Gyeonggido, Daegu, Gyeongsangbukdo and other areas). The annual income level in February 2020 was divided into quartiles. To reflect comorbidities, the Charlson Comorbidity Index (CCI) was calculated using ICD-10 codes from 2015 to 2019 (Table S1), which were used as confounders. In addition, data on the incidence of intracranial injury (S06) and thyroid disease (E01-E07) were extracted and considered as confounders. Since dementia is known to be closely related to underlying psychiatric illnesses [23], depression (F32, F33 and F34.1), substance use disorder (F10-19), anxiety disorder (F41) and post-traumatic stress disorder (PTSD; F43.1) were included as confounders in this study. Data regarding hospitalization, supplemental oxygen therapy, mechanical ventilator support and intensive care unit (ICU) admission were also collected and used as confounders.

### 2.7. Statistical Methodology

The clinicopathological characteristics of the total study population are presented as median values with interquartile range (IQR) and numbers with percentages for categorical variables. To compare the clinicopathological characteristics between COVID-19 survivors and the control group, a one-way analysis of variance and chi-square test were used for the CCI (continuous variable) and all other variables (categorical variables), respectively. First, we performed a univariable Cox regression analysis to examine whether COVID-19 survivors were individually associated with the development of dementia. In this time-to-event analysis, the diagnosis of dementia was set as the event and the duration from 1 January 2020 to the date of diagnosis of dementia was used as time. Next, we constructed four multivariable Cox regression models for the development of dementia and all covariates were included in the models for multivariable adjustment. In multivariable model 1, the association of COVID-19 survivors with the development of dementia was examined and compared to that of the control group. In multivariable model 2, COVID-19 survivors were divided into two groups according to the hospitalization status among COVID-19 survivors: hospitalized and non-hospitalized survivors. In multivariable model 3, COVID-19 survivors were divided into two groups according to the requirement for oxygen therapy (supplemental oxygen therapy and mechanical ventilator support). In multivariable model 4, the duration of COVID-19-related isolation was included as a continuous variable in the model. 

For Cox regression analyses for a specific type of dementia, the primary endpoint (overall dementia) was divided into three types of dementia: AD, VD and other types. For each of the three endpoints, three multivariable Cox regression models were constructed to investigate whether COVID-19 survivors had a higher risk of developing each type of dementia (AD, VD and others). For a sensitivity analysis, we performed a multivariable Cox regression analysis for the development of dementia after excluding test-negative individuals because of the potential for false negatives in the PCR test, which may have affected the results of this study [24]. Finally, subgroup analyses were performed using multivariable Cox regression modeling with respect to sex, age, CCI, underlying psychiatric illness, diabetes mellitus and cerebrovascular disease (CVD), which are all risk factors for the development of dementia [25]. Additionally, we performed a multivariable Cox regression analysis after excluding 8 patients in the control group who were diagnosed with COVID-19 between 4 June 2020 to 1 December 2020. In the subgroup analyses, all covariates were included in each multivariable Cox regression model except for the criterion based on which the subgroups were divided. For example, sex was excluded in the multivariable Cox regression model for the male subgroup analysis. The results of Cox regression analysis were presented as hazard ratios (HRs) with 95% confidence intervals (CIs) and the absence of multicollinearity between the variables in the multivariable models was confirmed when the variance inflation factor was <2.0. Log-log plots were used the confirm that the central assumption of Cox proportional hazard models was satisfied. R software (version 4.0.3; R Foundation for Statistical Computing, Vienna, Austria) was used for all analyses, except for stratified random sampling and a *p*-value < 0.05 was considered statistically significant. 

## 3. Results

**Figure 1:**
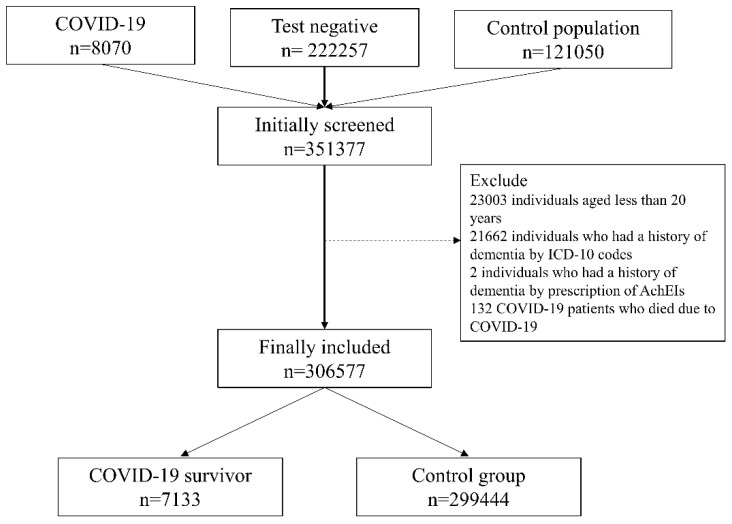
Flow chart depicting the study participant selection process. COVID-19, coronavirus disease-2019; ICD-10, International Classification of Diseases tenth revision; AchEIs, acetylcholinesterase inhibitors.

The NHIS-COVID-19 DB contained information on a total of 351,377 individuals compris-ing three groups (8070 COVID-19 patients, 222,257 individuals who tested negative and 121,050 in the control group). Among them, 23,004 individuals aged <20 years were ex-cluded from the analysis. Moreover, 21,664 individuals had a history of dementia accord-ing to ICD-10 codes (n = 21,662) and prescription of AchEIs (n = 2) were excluded. Next, 132 individuals who died due to COVID-19 during hospitalization were excluded from the analysis. Finally, 306,577 adults were included in the analysis, comprising 7133 COVID-19 survivors and 299,444 individuals in the control group, as shown in Figure 1. Table S2 shows the clinico-epidemiological characteristics of all the study participants. 

New-onset dementia, diagnosed in 2020, was recorded in 1.2% (3546 of 306,577) of adults, of which 3543 cases were diagnosed using the registered ICD-10 codes and three cases using AchEI prescription data. Among all cases of dementia, AD, VD and other types were present in 2668 cases (0.9%), 326 cases (0.1%) and 986 cases (0.3%), respectively. The results of the comparison of the clinicopathological characteristics between COVID-19 survivors and the control group are shown in Table 1. The proportion of male individuals was significantly higher in those in the control group (134,007/299,4444, 4.8%) than in the COVID-19 survivors (2813/7133, 39.4%) (*p* < 0.001). The proportion of individuals in the older age group (60–69, 70–79 and ≥80 years) was significantly higher (1103/7133, 15.5%; 467/7133, 6.5%; and 124/7133, 1.7%) than that of those in the control group (41,702/299,444, 13.9%; 41,702/299,444, 8.5%; and 15,082/299,444, 5.0%), respectively (*p* < 0.001).

### 3.1. Main Analyses

Table 2 presents the results of univariable and multivariable Cox regression analyses for the development of dementia. In covariate-adjusted model 1, the incidence of dementia in COVID-19 survivors was 1.39 times greater than that in adults who did not contract COVID-19. Other variables in multivariable model 1 are presented in Table S3. In covariate-adjusted model 2, COVID-19 survivors who were hospitalized for COVID-19 were associated with a 1.62-fold higher incidence of dementia than that of the control group, while COVID-19 survivors who were not hospitalized were not significantly associated with the development of dementia. In covariate-adjusted model 3, COVID-19 survivors who received oxygen therapy had 1.56 times the incidence of dementia in the control group. In covariate-adjusted model 4, a 1-day increase in the isolation duration due to COVID-19 was associated with a 1% higher incidence of dementia than that of the control group. 

### 3.2. Sensitivity Analysis after Excluding Test-Negative Individuals

Table S4 presents the results of the comparison of the clinicopathological characteristics between COVID-19 survivors and the control population after excluding test-negative individuals. The CCI was not significantly different for the COVID-19 survivors and the control population (*p* = 0.553). Table 3 shows the results of multivariable Cox regression analysis for the development of dementia after excluding test-negative individuals. The COVID-19 survivors had a 3.30-fold (HR: 3.30, 95% CI: 2.43–4.47; *p* < 0.001) higher incidence of dementia than that of the control population.

### 3.3. Specific Type of Dementia

Table 4 shows multivariable Cox regression models for the specific types of dementia. Compared to the control group, a statistically significant association between the incidence of dementia and COVID-19 survivorship was observed for AD (HR: 1.32, 95% CI: 1.05–1.86; *p* = 0.028) and other types of dementia (HR: 2.04, 95% CI: 1.25–3.32; *p* = 0.004), but not for VD (HR: 1.51, 95% CI: 0.62–3.70; *p* = 0.364).

### 3.4. Subgroup Analyses

Table 5 shows the results of the subgroup analyses. Stratified by sex, COVID-19 survivors in the female group showed a 1.56-fold higher (HR: 1.56, 95% CI: 1.08–2.25; *p* = 0.017) incidence of dementia than the control group. As for age, COVID-19 survivors in the older age group (≥60 years old) showed a 1.38-fold higher (HR: 1.38, 95% CI: 1.02–1.87; *p* = 0.039) incidence of dementia than the control group. According to the CCI, COVID-19 survivors in the CCI ≥3 group showed 1.42 times (HR: 1.42, 95% CI: 1.00–1.91; *p* = 0.048) the incidence of dementia in the control group. Moreover, COVID-19 survivors with diabetes or CVD showed 1.52 (HR: 1.52, 95% CI: 1.06–2.54; *p* = 0.022) and 1.64 times (HR: 1.64, 95% CI: 1.06–2.54; *p* = 0.024) the incidence of dementia in the control group, respectively. After excluding 8 patients in the control group who were diagnosed with COVID-19 between 4 June 2020 to 1 December 2020, the incidence of dementia in COVID-19 survivors was 1.39 times (HR: 1.39, 95% CI: 1.04–1.85; *p* = 0.024) that of the control group.

## 4. Discussion

Using a nationwide population-based database in South Korea, COVID-19 survivors were shown to be associated with a higher incidence of overall dementia and this association was more evident in COVID-19 survivors who were admitted to hospital due to COVID-19 or who underwent oxygen therapy. Moreover, the association between dementia and COVID-19 survivorship was statistically significant for AD and other types of dementia, but not for VD. In the subgroup analyses, the increased association of dementia was more evident in the female group, older age group (≥60 years old), CCI ≥ 3 group and those with underlying diabetes and CVD among the COVID-19 survivors. The results of this study suggest that COVID-19 survivors with these risk factors may have a increased risk for dementia, especially AD and other types of dementia, at approximately 6 months’ follow-up period. Considering that COVID-19 has been suggested as a risk factor for the progression to dementia, especially AD [26], this is the first report to deduce that COVID-19 can be a risk factor for the diagnosis of dementia, such as AD, among survivors at 6 months’ follow-up.

A few possible mechanisms have been suggested to explain the effect of COVID-19 on the AD diagnosis in this study. COVID-19 is associated with a severe immune response and an increase in systemic cytokine levels [13]; systemic inflammation has been shown to promote cognitive decline and contribute to the pathogenesis of neurodegenerative diseases such as AD [27]. Moreover, SARS-CoV-2 also invades the CNS and is associated with neuroinflammation [28]. Previous studies have reported that SARS-CoV-2 directly causes viral encephalitis and SARS-CoV-2 was detected in the cerebrospinal fluid of patients, demonstrating the neuroinvasive potential of SARS-CoV-2 [29]. In the process of neuroinflammation, the coronavirus ORF3a protein is known to activate the NLRP3 inflammasome [30]. In turn, NLRP3 inflammasome-mediated neuroinflammation adversely affects beneficial immune functions in the brain and causes the pathological accumulation of neurodegeneration-associated peptides, such as fibrillar amyloid-β [31]. The pathological accumulation of amyloid-β peptide is known to be an important mechanism in the pathogenesis of AD [32]. In addition, activation of the NLRP3 inflammasome is known to directly induce or aggravate neurodegenerative processes that lead to functional impairment in AD [33]. 

In this study, COVID-19 survivors who were admitted to a hospital due to COVID-19 or who underwent oxygen therapy were associated with an increased risk of dementia, while those with no symptoms or mild symptoms were not, suggesting that the disease severity of COVID-19 might have affected the results. ICU admission and invasive treatment such as mechanical ventilator care for acute respiratory distress syndrome (ARDS) are known risk factors for cognitive decline [34]. In the current pandemic, due to SARS-CoV-2, a retrospective cohort study of 1591 COVID-19 patients admitted to the ICU in Italy showed that 88% of COVID-19 patients required mechanical ventilation [35]. Moreover, COVID-19 survivors who required oxygen therapy might have a higher viral load of SARS-CoV-2 than those with mild or no symptoms. This suggests that COVID-19 survivors who required hospitalization or oxygen therapy might have a higher possibility of sequalae such as neurodegenerative diseases, including AD [36].

The results of the subgroup analyses are also novel. Our study revealed that COVID-19 survivors with a higher underlying risk of dementia, due to diabetes, old age, female sex, higher CCI score and CVD were at a significantly higher risk of dementia. The risk factors for dementia diagnosis and COVID-19, such as old age and comorbidity status, are known to overlap [37]. Our study also reveals that COVID-19 survivors with an underlying risk of dementia are more vulnerable to the development of dementia [38]. For example, comorbidities, such as diabetes and cerebrovascular disease, were known risk factors for dementia, as our study reported similarly in COVID-19 survivors [39]. 

The HR of COVID-19 survivors for the risk of dementia was higher after excluding test-negative individuals in this study. In Table 1, the CCI was significantly higher in the control group (control population and test-negative individuals), while CCI did not significantly differ between COVID-19 survivors and the control population after excluding test-negative individuals as shown in Table S4. In South Korea, after extensive contact tracing for all COVID-19 patients before diagnosis by PCR test, the KDCPA tested all those individuals for COVID-19 who had been in direct or indirect contact with COVID-19 patients in the community or hospital [20]. Therefore, several older individuals had to be tested by PCR due to contact with with their children who were more active than the older people and were thus more likely to be test-positive or come into contact with COVID-19 patients. Moreover, if a patient in certain long-term facility care centers is diagnosed with COVID-19, all patients in the center undergo PCR testing for early detection of COVID-19, because they are a high-risk group for COVID-19 related death [40]. Therefore, the test-negative group comprised older patients with comorbidities and including these patients led to a higher prevalence of comorbidities in the control group as shown in Table 1.

Our study had some limitations. First, important parameters related to dementia risks, such as the body mass index, alcohol consumption and smoking history, were not included in this study because the NHIS database did not contain the relevant data. Smoking and alcohol use are known risk factors for the dementia diagnosis [41], the absence of which may have affected our results. Second, while we used multivariable adjustment with known and measured confounders, unmeasured and unknown confounders may have affected the results. For example, some comorbidities, such as hypertension or atrial fibrillation, were not adjusted for as confounders and it might have affected the results in this study. Third, the severity of dementia was not evaluated in this study because of the limitations of the data source. Fourth, although treatment information such as that for oxygen therapy and hospital admission among COVID-19 survivors was used, the severity of the infection was not reflected accurately through the laboratory results. Fifth, although all patients with dementia should be registered in the NHIS DB to receive financial support for treatment expenses, some cases of dementia may have been missed. For example, some individuals with mild dementia symptoms might not visit the outpatient clinic for diagnosis, which may affect the results of this study. Lastly, there was a medical surveillance bias in this study that affected the main result. As individuals who have undergone testing, as well as those who have been confirmed of having been tested, are more likely to be under medical surveillance, such individuals are more likely to be tested and be diagnosed with dementia. The sensitivity analysis with removing those who tested negative for COVID-19 in Table 4 supports this medical surveillance bias. In other words, the HR in the sensitivity analysis tends to be higher than that in the main analysis, suggesting that individuals with positive or negative PCR test results are more likely diagnosed with dementia.

## 5. Conclusions

In summary, at approximately 6 months’ follow-up, we showed that COVID-19 survivors were at a higher risk of new-onset dementia in South Korea and this association was more evident in survivors who were admitted to the hospital for COVID-19 or who required oxygen therapy. Among COVID-19 survivors, the increased association of dementia was more evident in females, older adults (≥60 years old), those with a CCI of ≥3 and those with underlying diabetes or CVD. Moreover, the increased risk of dementia in COVID-19 survivors was significant for AD and other types of dementia, but not for VD. Further research should be performed to evaluate the long-term sequalae of dementia among COVID-19 survivors to confirm our findings.

## Figures and Tables

**Table 1:** The comparison of the clinicopathological characteristics between COVID-19 survivors and the control group.

Variable		COVID-19 Survivor n = 7133	Control Group n = 299,444	*p*-Value
Sex: male		2813 (39.4)	134,007 (44.8)	<0.001
Age				<0.001
	20–29	2057 (28.8)	68,314 (22.8)	
	30–39	829 (11.6)	50,378 (16.8)	
	40–49	1030 (14.4)	46,706 (15.6)	
	50–59	1523 (21.4)	51,882 (17.3)	
	60–69	1103 (15.5)	41,702 (13.9)	
	70–79	467 (6.5)	25,380 (8.5)	
	≥80	124 (1.7)	15,082 (5.0)	
Residence in 2020				<0.001
	Seoul	500 (7.0)	49,906 (16.7)	
	Gyeonggido	407 (5.7)	53,104 (17.7)	
	Daegu	4673 (65.5)	92,507 (30.9)	
	Gyeongsangbukdo	790 (11.1)	24,119 (8.1)	
	Other area	763 (10.7)	79,808 (26.7)	
Annual income level in 2020				<0.001
	Q1 (lowest)	2204 (30.9)	67,301 (22.5)	
	Q2	1390 (19.35)	60,106 (20.1)	
	Q3	1467 (20.6)	73,800 (24.6)	
	Q4	1960 (27.5)	93,035 (31.1)	
	Unknown	112 (1.6)	5202 (1.7)	
Charlson comorbidity index		2.3 (2.3)	3.0 (3.0)	<0.001
	Myocardial infarction	158 (2.2)	9114 (3.0)	<0.001
	Congestive heart failure	324 (4.5)	26,841 (9.0)	<0.001
	Peripheral vascular disease	1050 (14.7)	53,536 (17.9)	<0.001
	Cerebrovascular disease	599 (8.4)	34,785 (11.6)	<0.001
	Chronic pulmonary disease	3565 (50.0)	170,197 (56.8)	<0.001
	Rheumatic disease	657 (9.2)	31,154 (10.4)	0.001
	Peptic ulcer disease	2848 (39.9)	137,331 (45.9)	<0.001
	Mild liver disease	2960 (41.5)	134,913 (45.1)	<0.001
	DM without chronic complication	1522 (21.3)	79,194 (26.4)	<0.001
	DM with chronic complication	429 (6.0)	25,168 (8.4)	<0.001
	Hemiplegia or paraplegia	67 (0.9)	3572 (1.2)	0.051
	Renal disease	131 (1.8)	12,355 (4.1)	<0.001
	Any malignancy	510 (7.1)	42,191 (14.1)	<0.001
	Moderate or severe liver disease	24 (0.3)	2576 (0.9)	<0.001
	Metastatic solid tumour	58 (0.8)	8228 (2.7)	<0.001
	AIDS/HIV	7 (0.1)	551 (0.2)	0.093
	Intracranial Injury	4 (0.1)	437 (0.1)	0.069
	Thyroid disorder	2310 (32.4)	108,355 (36.2)	<0.001
Underlying psychiatric illness				
	Anxiety disorder	1461 (20.5)	72,405 (24.2)	<0.001
	Substance abuse	96 (1.3)	5091 (1.7)	0.022
	Depression	971 (13.6)	49,016 (16.4)	<0.001
	PTSD	11 (0.2)	520 (0.2)	0.696
Hospital admission in 2020		797 (11.2)	38,745 (12.9)	<0.001
Supplemental Oxygen therapy		758 (10.6)	37,036 (12.4)	<0.001
Mechanical ventilator support		72 (1.0)	5359 (1.8)	<0.001
ICU admission in 2020		162 (2.3)	11,814 (3.9)	<0.001
Development of dementia		49 (0.7)	3497 (1.2)	<0.001
	Alzheimer’s dementia	36 (0.5)	2632 (0.9)	0.001
	Vascular dementia	5 (0.1)	321 (0.1)	0.342
	Other dementia	17 (0.2)	969 (0.3)	0.209

Control group: adults who did not contract COVID-19. Presented as median value with IQR for Charlson comorbidity index and number with percentage for other variables (categorical variables) COVID-19, coronavirus disease, DM, diabetes mellitus; AIDS, acquired immunodeficiency syndrome; HIV, human immunodeficiency virus; PTSD, post-traumatic stress disorder; IQR, interquartile range.

**Table 2:** Univariable and multivariable Cox regression analyses for the development of dementia.

Variable		Cox Regression Analysis	*p*-Value
		HR (95% CI)	
Unadjusted (univariable analysis)			
	COVID-19 survivors (vs. Control group)	0.59 (0.44, 0.78)	<0.001
Covariates-adjusted model 1 (multivariable analysis)			
	COVID-19 survivors (vs. Control group)	1.39 (1.05, 1.85)	0.023
Covariates-adjusted model 2 (multivariable analysis)			
	Control group (n = 299,444)	1	
	COVID-19 survivors without hospitalization (n = 6336)	1.28 (0.89, 1.84)	0.185
	COVID-19 survivors with hospitalization (n = 797)	1.62 (1.03, 2.54)	0.038
Covariates-adjusted model 3 (multivariable analysis)			
	Control group (n = 299,444)	1	
	COVID-19 survivors without oxygen therapy (n = 6375)	1.31 (0.92, 1.87)	0.139
	COVID-19 survivors with oxygen therapy (n = 758)	1.56 (1.02, 2.48)	0.042
Covariates-adjusted model 4 (multivariable analysis)			
	Duration of isolation due to COVID-19, day	1.01 (1.00, 1.02)	0.011

Control group: adults who did not contract COVID-19. Other variables in the multivariable model 1 are presented in Table S3. HR, hazard ratio; CI, confidence interval; COVID-19, coronavirus disease.

**Table 3:** Multivariable Cox regression analysis for development of dementia after excluding test negative individuals.

Variable		Cox Regressuib Analysis	*p*-Value
		HR (95% CI)	
Covariates-adjusted model 1 (multivariable analysis)			
	Control group	1	
	COVID-19 survivors	3.30 (2.43, 4.47)	<0.001
Covariates-adjusted model 2 (multivariable analysis)			
	Control group (n = 299,444)	1	
	COVID-19 survivors without hospitalization (n = 6336)	3.09 (2.11, 4.52)	<0.001
	COVID-19 survivors with hospitalization (n = 797)	3.68 (2.31, 5.88)	<0.001
Covariates-adjusted model 3 (multivariable analysis)			
	Control group (n = 299,444)	1	
	COVID-19 survivors without oxygen therapy (n = 6375)	3.17 (2.18, 4.62)	<0.001
	COVID-19 survivors with oxygen therapy (n = 758)	3.53 (2.19, 5.71)	<0.001
Covariates-adjusted model 4 (multivariable analysis)			
	Duration of isolation due to COVID-19, day	1.03 (1.02, 1.03)	<0.001

Control group: adults who did not contract COVID-19. HR, hazard ratio; CI, confidence interval; COVID-19, Coronavirus disease.

**Table 4:** Multivariable Cox regression models for specific type of dementia.

Variable		Multivariable Model	*p*-Value
		HR (95% CI)	
Alzheimer’s dementia			
	COVID-19 survivors (vs. control group)	1.32 (1.05, 1.86)	0.028
Vascular dementia			
	COVID-19 survivors (vs. control group)	1.51 (0.62, 3.70)	0.364
Other dementia			
	COVID-19 survivors (vs. control group)	2.04 (1.25, 3.32)	0.004

Control group: adults who did not contract COVID-19. HR, hazard ratio; CI, confidence interval; COVID-19, coronavirus disease.

**Table 5:** Subgroup analyses.

Variable		Dementia Event (%)	Multivariable Model	*p*-Value
			HR (95% CI)	
Male (n = 136,820)				
	Control group	1687 (1.3)	1	
	COVID-19 survivors	19 (0.7)	1.23 (0.78, 1.94)	0.376
Female (n = 169,757)				
	Control group	1809 (1.1)	1	
	COVID-19 survivors	30 (0.7)	1.56 (1.08, 2.25)	0.017
Age: 20–59 (n = 222,719)				
	Control group	248 (0.1)	1	
	COVID-19 survivors	6 (0.1)	1.72 (0.76, 3.92)	0.197
Age: ≥ 60 (n = 83,858)				
	Control group	3248 (4.0)	1	
	COVID-19 survivors	43 (2.5)	1.38 (1.02, 1.87)	0.039
CCI: 0–2 (n = 173,189)				
	Control group	541 (0.3)	1	
	COVID-19 survivors	11 (0.2)	1.38 (0.76, 2.54)	0.293
CCI: ≥ 3 (n = 133,388)				
	Control group	2955 (2.3)	1	
	COVID-19 survivors	38 (1.5)	1.42 (1.00, 1.91)	0.048
Underlying anxiety disorder (n = 73,866)				
	Control group	1701 (2.3)	1	
	COVID-19 survivors	19 (1.3)	1.10 (0.70, 1.73)	0.690
Underlying depression group (n = 49,987)				
	Control group	1264 (2.6)	1	
	COVID-19 survivors	19 (2.0)	1.48 (0.94, 2.35)	0.094
Underlying PTSD group (n = 531)				
	Control group	5 (1.0)	1	
	COVID-19 survivors	0 (0.0)	0.00 (0.00)	0.824
Underlying substance abuse (n = 5187)				
	Control group	113 (2.2)	1	
	COVID-19 survivors	3 (3.1)	1.77 (0.52, 5.97)	0.358
Underlying diabetes group (n = 84,807)				
	Control group	2319 (2.8)	1	
	COVID-19 survivors	31 (1.9)	1.52 (1.06, 2.18/)	0.022
Underlying CVD group (n = 35,385)				
	Control group	1468 (4.2)	1	
	COVID-19 survivors	21 (3.5)	1.64 (1.06, 2.54)	0.024
After excluding 8 patients in the control group, who were diagnosed as COVID-19 from 4 June 2020 to 1 December 2020				
	Control group	3496 (1.2)	1	
	COVID-19 survivors	49 (0.7)	1.39 (1.04, 1.85)	0.024

Control group: adults who did not contract COVID-19. HR, hazard ratio; CI, confidence interval; COVID-19, coronavirus disease; PTSD, post-traumatic stress disorder; CVD, cerebrovascular disease.

## Data Availability

Data will be available upon reasonable request to corresponding author.

## Supplementary Materials

The following are available online at https://www.mdpi.com/article/10.3390/jpm11101015/s1, Table S1: The ICD-10 codes used by comorbidity to compute the Charlson comorbidity index, Table S2: The clinico-epidemiological characteristics of all the study participants (n=306,577), Table S3: Other variables in multivariable model 1 from Table 2, Table S4: The comparison of the clinicopathological characteristics between COVID-19 survivors and the control population after excluding test-negative individuals.
