# Supplementary material for: Dementia Risk among Coronavirus Disease Survivors: A Nationwide Cohort Study in South Korea
Source: J Pers Med. 2021 Oct 9;11(10):1015. doi: 10.3390/jpm11101015 (PMC8540001; doi:10.3390/jpm11101015)
Supplement: Supplementary file 1 [file jpm-11-01015-s001.zip › Table S2_rev.pdf]

Table S2. The clinico-epidemiological characteristics of all the study participants (n=306,577)

Variable	Number (%)	Mean (SD)
Sex: male	136,820 (44.6)	
Age		
20-29	70,371 (23.0)	
30-39	51,207 (16.7)	
40-49	47,736 (15.6)	
50-59	53,405 (17.4)	
60-69	42,805 (14.0)	
70-79	25,847 (8.4)	
≥80	15,206 (5.0)	
Residence in 2020		
Seoul	50,406 (16.4)	
Gyeonggido	53,511 (17.5)	
Daegu	97,180 (31.7)	
Gyeongsangbukdo	24,909 (8.1)	
Other area	80,571 (26.3)	
Annual income level in 2020		
Q1 (lowest)	69,505 (22.7)	
Q2	61,496 (20.1)	
Q3	75,267 (24.6)	
Q4	94,995 (31.0)	
Unknown	5,314 (1.7)	
Charlson comorbidity index		3.0 (3.0)
Myocardial infarction	9,272 (3.0)	
Congestive heart failure	27,165 (8.9)	
Peripheral vascular disease	54,586 (17.8)	

Cerebrovascular disease	35,384 (11.5)
Chronic pulmonary disease	173,762 (56.7)
Rheumatic disease	31,811 (10.4)
Peptic ulcer disease	140,179 (45.7)
Mild liver disease	137,873 (45.0)
Diabetes without chronic complication	80,716 (26.3)
Diabetes with chronic complication	25,597 (8.3)
Hemiplegia or paraplegia	3,639 (1.2)
Renal disease	12,486 (4.1)
Any malignancy	42,701 (13.9)
Moderate or severe liver disease	2,600 (0.8)
Metastatic solid tumour	8,286 (2.7)
AIDS/HIV	558 (0.2)
Intracranial Injury	441 (0.1)
Thyroid disorder	110,665 (36.1)
Underlying psychiatric illness	
Anxiety disorder	73,866 (24.1)
Substance disorder	5,187 (1.7)
Depression	49,987 (16.3)
PTSD	531 (0.2)
Hospital admission in 2020	39,542 (12.9)
Supplemental Oxygen therapy	37,794 (12.3)
Mechanical ventilator support	5,431 (1.8)
ICU admission in 2020	11,976 (3.9)
Development of dementia	3,546 (1.2)
Alzheimer’s dementia	2,668 (0.9)
Vascular dementia	326 (0.1)

Other dementia	986 (0.3)

SD, standard deviation; AIDS, acquired immunodeficiency syndrome; HIV, human immunodeficiency virus; PTSD, post-traumatic stress disorder
