# Supplementary material for: Dementia Risk among Coronavirus Disease Survivors: A Nationwide Cohort Study in South Korea
Source: J Pers Med. 2021 Oct 9;11(10):1015. doi: 10.3390/jpm11101015 (PMC8540001; doi:10.3390/jpm11101015)
Supplement: Supplementary file 1 [file jpm-11-01015-s001.zip › Table S3_rev.pdf]

Table S3. Other variables in multivariable model 1 from Table 2

Other variables in model 1	Multivariable model<br>HR (95% CI)	*P*-value
Sex: male	0.95 (0.88, 1.01)	0.109
Age, 10-year increase	3.00 (2.90. 3.11)	<0.001
Residence in 2020		
Seoul	1	
Gyeonggido	0.65 (0.59, 0.71)	<0.001
Daegu	0.80 (0.73, 0.87)	<0.001
Gyeongsangbukdo	0.32 (0.29, 0.36)	<0.001
Other area	0.59 (0.53, 0.67)	<0.001
Annual income level in 2020		
Q1 (lowest)	1	
Q2	1.22 (0.92, 1.64)	0.171
Q3	0.92 (0.68, 1.24)	0.579
Q4	0.90 (0.67, 1.21)	0.486
Unknown	0.86 (0.64, 1.15)	0.299
Charlson comorbidity index	1.19 (1.06, 1.33)	0.003
Myocardial infarction	1.08 (0.96, 1.20)	0.194
Congestive heart failure	1.18 (1.09, 1.27)	<0.001
Peripheral vascular disease	1.09 (1.02, 1.17)	0.016
Cerebrovascular disease	1.29 (1.20, 1.39)	<0.001
Chronic pulmonary disease	0.86 (0.79, 0.93)	<0.001
Rheumatic disease	0.94 (0.86, 1.02)	0.153
Peptic ulcer disease	0.91 (0.84, 0.97)	0.007
Mild liver disease	0.97 (0.90, 1.05)	0.512

Diabetes without chronic complication	1.13 (1.04, 1.22)	0.003
Diabetes with chronic complication	1.13 (1.04, 1.23)	0.003
Hemiplegia or paraplegia	1.79 (1.57, 2.05)	<0.001
Renal disease	1.19 (1.08, 1.31)	<0.001
Any malignancy	0.88 (0.81, 0.95)	0.002
Moderate or severe liver disease	1.31 (1.04, 1.65)	0.022
Metastatic solid tumour	0.96 (0.84, 1.11)	0.605
AIDS/HIV	1.99 (1.23, 3.21)	0.005
Intracranial Injury	1.54 (0.93, 2.56)	0.095
Thyroid disorder	0.90 (0.83, 0.97)	0.024
Underlying psychiatric illness		
Anxiety disorder	1.20 (1.11, 1.29)	<0.001
Substance disorder	1.85 (1.53, 2.24)	<0.001
Depression	1.23 (1.14, 1.32)	<0.001
PTSD	1.45 (0.60, 3.48)	0.410

HR, hazard ratio; CI, confidence interval; AIDS, acquired immunodeficiency syndrome; HIV, human immunodeficiency virus; PTSD, post-traumatic stress disorder
