# Supplementary material for: Dementia Risk among Coronavirus Disease Survivors: A Nationwide Cohort Study in South Korea
Source: J Pers Med. 2021 Oct 9;11(10):1015. doi: 10.3390/jpm11101015 (PMC8540001; doi:10.3390/jpm11101015)
Supplement: Supplementary file 1 [file jpm-11-01015-s001.zip › Table S4.pdf]

Table S4. The comparison of the clinicopathological characteristics between COVID-19 survivors and the control population after excluding test-negative individuals

Variable	COVID-19 survivor n=7,133	Control population n=111,825	P-value
Sex: male	2,813 (39.4)	44,577 (39.9)	0.475
Age			<0.001
20-29	2,057 (28.8)	30,843 (27.6)	
30-39	829 (11.6)	12,472 (11.2)	
40-49	1,030 (14.4)	15,514 (13.9)	
50-59	1,523 (21.4)	23,329 (20.9)	
60-69	1,103 (15.5)	17,511 (15.7)	
70-79	467 (6.5)	8,197 (7.3)	
80+	124 (1.7)	3,959 (3.5)	
Residence in 2020			0.427
Seoul	500 (7.0)	7,554 (6.8)	
Gyeonggido	407 (5.7)	6,274 (5.6)	
Daegu	4,673 (65.5)	73,128 (65.4)	
Gyeongsangbukdo	790 (11.1)	13,161 (11.8)	
Other area	763 (10.7)	11,708 (10.5)	
Annual income level in 2020			<0.001
Q1 (lowest)	2,204 (30.9)	28,303 (25.3)	
Q2	1,390 (19.35)	23,115 (20.7)	
Q3	1,467 (20.6)	25,647 (22.9)	
Q4	1,960 (27.5)	32,823 (29.4)	
Unknown	112 (1.6)	1,937 (1.7)	
Charlson comorbidity index	2 [1,3]	2 [1,3]	0.553

Myocardial infarction	158 (2.2)	2,103 (1.9)	0.045
Congestive heart failure	324 (4.5)	5,472 (4.9)	0.182
Peripheral vascular disease	1,050 (14.7)	17,341 (15.5)	0.075
Cerebrovascular disease	599 (8.4)	9,766 (8.7)	0.330
Chronic pulmonary disease	3,565 (50.0)	53,971 (48.3)	0.005
Rheumatic disease	657 (9.2)	9,486 (8.5)	0.033
Peptic ulcer disease	2,848 (39.9)	47,024 (42.1)	<0.001
Mild liver disease	2,960 (41.5)	45,207 (40.4)	0.074
Diabetes without chronic complication	1,522 (21.3)	24,061 (21.5)	0.721
Diabetes with chronic complication	429 (6.0)	6944 (6.2)	0.507
Hemiplegia or paraplegia	67 (0.9)	737 (0.7)	0.005
Renal disease	131 (1.8)	2,408 (2.2)	0.073
Any malignancy	510 (7.1)	8,673 (7.8)	0.063
Moderate or severe liver disease	24 (0.3)	390 (0.3)	0.864
Metastatic solid tumour	58 (0.8)	919 (0.8)	0.937
AIDS/HIV	7 (0.1)	127 (0.1)	0.973
Intracranial Injury	4 (0.1)	107 (0.1)	0.288
Thyroid disorder	2,310 (32.4)	35,176 (31.5)	0.102
Underlying psychiatric illness			
Anxiety disorder	1,461 (20.5)	23,192 (20.7)	0.603
Substance disorder	96 (1.3)	1,068 (1.0)	0.001
Depression	971 (13.6)	13,966 (12.5)	0.007
PTSD	11 (0.2)	147 (0.1)	0.609
Hospital admission in 2020	797 (11.2)	1,515 (1.4)	<0.001
Supplemental Oxygen therapy	758 (10.6)	1,467 (1.3)	<0.001
Mechanical ventilator support	72 (1.0)	56 (0.1)	<0.001
ICU admission in 2020	162 (2.3)	200 (0.2)	<0.001

Development of dementia	49 (0.7)	312 (0.3)	<0.001
Alzheimer's dementia	36 (0.5)	242 (0.2)	<0.001
Vascular dementia	5 (0.1)	21 (0.0)	0.004
Other dementia	17 (0.2)	66 (0.1)	<0.001

Presented as median value with IQR for Charlson comorbidity index, and number with percentage for other variables (categorical variables)

COVID-19, coronavirus disease, DM, diabetes mellitus; AIDS, acquired immunodeficiency syndrome; HIV, human immunodeficiency virus; PTSD, post-traumatic stress disorder; IQR, interquartile range
